# The value of the neutrophil to lymphocyte ratio and PLT count for the diagnosis and prediction of COVID-19 severity

**DOI:** 10.1371/journal.pone.0293432

**Published:** 2023-10-30

**Authors:** Yingji Chen, Pingyang Han, Yunjie Gao, Ruifeng Jiang, Mei Tao, Ximin Li

**Affiliations:** Department of Nephrology, The Affiliated Hospital of Hangzhou Normal University, Hangzhou, China; Azienda Ospedaliero Universitaria Careggi, ITALY

## Abstract

**Background:**

COVID-19 and influenza A can cause severe respiratory illness. Differentiating between the two diseases and identifying critically ill patients in times of epidemics become a challenge for frontline medical staff. We sought to investigate whether both diseases and their severity could be recognized by routine blood parameters.

**Methods:**

Our retrospective study analysed the clinical data and first-time routine blood parameters of 80 influenza A patients and 123 COVID-19 patients. COVID-19 patients were divided into three groups according to treatment modalities and outcomes: outpatient group, inpatient without invasive mechanical ventilation (IMV) group, and inpatient with IMV group. We used the Mann-Whitney and Kruskal-Wallis tests to analyze the differences in routine blood parameters between the two or three groups. Receiver operating characteristic (ROC) curve analysis and area under the curve (AUC) were used to assess the diagnostic accuracy.

**Results:**

Compared with outpatient influenza A patients, outpatient COVID-19 patients had a higher neutrophil to lymphocyte ratio (NLR) (6.63 vs 3.55). ROC analysis showed that the NLR had a high diagnostic value for differentiating COVID-19 from influenza A (AUC = 0.739). The best cut-off point of the NLR was 6.48, the diagnostic sensitivity was 0.523, and the specificity was 0.925. The median platelet (PLT) count in the different COVID-19 groups was as follows: outpatient group (189×109/L), inpatient without IMV group (161×109/L), and inpatient with IMV group (94×109/L). Multivariate logistic regression analysis found a significant association between PLT and treatment modality and outcome in COVID-19 patients (p<0.001).

**Conclusions:**

NLR can be used as a potential biological indicator to distinguish COVID-19 and influenza A. Decreased PLT predicts the critical condition of COVID-19 patients and helps stratify the treatment of COVID-19 patients.

## Introduction

Coronavirus disease 2019 (COVID-19) and influenza A can cause viral pneumonia through respiratory transmission. Fever, cough, and headache are common clinical manifestations [1, 2]. Reverse transcription polymerase chain reaction (RT‒PCR) is the gold standard for the diagnosis of COVID-19 and influenza A, with expensive and time-consuming drawbacks, and it may miss 50% of COVID-19 cases by nose and throat swab polymerase chain reaction [3]. COVID-19 can cause acute respiratory distress syndrome (ARDS) and high mortality [4], and the treatment is very different from influenza A. Diagnosing COVID-19 and influenza A by routine laboratory tests (e.g., routine blood parameters) and identifying patients with COVID-19 who require IMV is necessary. The routine blood test is a commonly used laboratory test in the clinic because it is inexpensive and rapid, especially in febrile patients. This study provides a reliable reference for the diagnosis and prognosis of COVID-19 disease potential laboratory markers, by retrospectively analysing various parameters in the routine blood of COVID-19 and influenza A patients.

## Methods

### Study population

This retrospective study included 80 influenza A patients and 123 COVID-19 patients who were diagnosed at the Affiliated Hospital of Hangzhou Normal University from December 2021 to January 2022 and from December 2022 to January 2023. All COVID-19 and influenza A cases were diagnosed by nose and throat swab polymerase chain reaction (PCR). In the period between 29 April 2023 to 14 May 2023, we collected and analysed the first blood test data and clinical results of patients after the onset of illness through the hospital medical record network system. All included individuals were excluded from hematologic systemic diseases, liver cirrhosis, and chronic obstructive pulmonary disease, which can cause abnormalities in blood cells or require oxygen therapy According to the treatment modality and outcome, COVID-19 patients were divided into an outpatient group, an inpatient without IMV group, and an inpatient with IMV group. All influenza A cases were outpatient patients.

### Statistical analyses

Data were evaluated in IBM SPSS Statistics 25.0 (IBM Corp.) statistical software. Blood count data are expressed as 10^9^/L. Data are expressed as the median (P25, P75). The Shapiro-Wilk test was used to assess whether data were normally or nonnormally distributed. A t-test or Mann-Whitney U test was used to compare differences between two groups, and the Kruskal-Wallis test was used to compare medians among three groups. Categorical variables were compared between two groups using Pearson’s χ^2^ or Fisher’s exact test. Single‐factor parameters (p<0.05) were included in binary logistic regression analysis or multiple logistic regression analysis, and receiver operating characteristic (ROC) curves were used to diagnose disease efficacy. Statistical significance was defined as p<0.05.

## Results

### Patient characteristics

This study included 123 COVID-19 patients and 80 influenza A patients. The median age of outpatient COVID-19 group (n = 88) was 31 years (Interquartile range, IQR: 23–45), inpatient without IMV group (n = 17) was 67 years (IQR: 60–68), and inpatient with IMV group (n = 18) was 84 years (IQR: 82–87). The mean age of inpatients with IMV COVID-19 was older than that of the other two COVID-19 groups, but there was no significant difference between inpatients with IMV COVID-19 and inpatients without IMV (P = 0.546). The influenza A group was all outpatients, and the median age was 33 years (IQR: 29–37), which was not significantly different compared to the outpatient COVID-19 group (p = 0.212) (Tables 1 and 2).

**Table 1:** Various routine blood parameters of the outpatient COVID-19 and outpatient influenza A groups.

	COVID-19 (Outpatient)	Influenza A (0utpatient)	*P* value
NO.	88	80	
**Age, y**	31(23–45)	33(29–37)	.212
**Male (%)**	38 (43.2)	34 (42.5)	.929
**Female (%)**	50 (56.8)	46 (57.5)	
**WBC(×10** ^**9**^ **/L)**	6.43 (4.95–7.91)	5.46 (4.55–6.87)	.004
**NE(×10** ^**9**^ **/L)**	5.13 (3.58–6.28)	3.57 (2.88–4.87)	<.001
**NE%**	77.50 (67.72–82.82)	70.90 (62.72–74.97)	<.001
**LY(×10** ^**9**^ **/L)**	0.74 (0.48–1.06)	0.99 (0.81–1.33)	<.001
**LY%**	11.90 (7.50–17.77)	19.10 (15.45–25.87)	<.001
**MO(×10** ^**9**^ **/L)**	0.61 (0.48–0.79)	0.50 (0.40–0.61)	<.001
**MO%**	10.4 (7.7–12.9)	9.1 (7.3–10.7)	.018
**EO(×10** ^**9**^ **/L)**	0.015 (0.000–0.050)	0.020 (0.010–0.040)	.612
**EO%**	0.2 (0.1–0.7)	0.4 (0.1–0.7)	.147
**PLT(×10** ^**9**^ **/L)**	189 (160–225)	197 (164–221)	.770
**CRP (mg/L)**	7.91 (3.29–14.66)	7.41 (3.82–12.79)	.997
**NLR**	6.63 (3.84–10.97)	3.55 (2.42–4.86)	<.001
**LCR**	0.087 (0.051–0.224)	0.157 (0.077–0.243)	.019
**CLR**	11.49 (4.44–19.58)	6.33 (4.10–12.87)	.019

Note: Data are expressed as medians (P25, P75) or numbers (%).

Abbreviations: WBC, white blood cell; NE, neutrophil count; NE%, percentage of neutrophils; LY, lymphocyte count; LY%, percentage of lymphocytes; MO, monocyte count; MO%, percentage of monocytes; EO, eosinophil count; EO%, percentage of eosinophils; PLT, platelet count; NLR, neutrophil to lymphocyte ratio; LCR, lymphocyte to CRP ratio; CLR, CRP to lymphocyte ratio.

**Table 2:** Various routine blood parameters of the overall COVID-19, outpatient COVID-19, inpatient without IMV COVID-19, and inpatient with IMV COVID-19 groups.

	Overall	Outpatient	Inpatient without IMV	Inpatient with IMV	*P* ^1^	*P* ^2^	*P* ^3^
**NO.**	123	88	17	18			
**Age, y**	41(26–67)	31(23–45)	67(60–68)	84(82–87)	.000	.000	.546
**Male (%)**	60(48.8)	38 (43.2)	8(47.1)	14(77.8)	.596	.118	.164
**Female (%)**	63(51.2)	50 (56.8)	9(52.9)	4(22.2)			
**WBC(×10** ^**9**^ **/L)**	5.81(4.67–7.26)	6.43 (4.95–7.91)	4.86(4.03–6.68)	5.05(3.31–10.06)	.205		
**NE(×10** ^**9**^ **/L)**	4.20(3.23–5.74)	5.13 (3.58–6.28)	3.81(2.72–5.41)	4.20(2.55–9.15)	.384		
**NE%**	73.35(65.6–79.9)	77.50 (67.72–82.82)	74.80(69.25–81.00)	89.00(83.20–92.32)	1.0	.000	.000
**LY(×10** ^**9**^ **/L)**	0.91(0364–1.14)	0.74 (0.48–1.06)	0.74(0.65–0.91)	0.38(0.24–0.54)	1.0	.000	.000
**LY%**	16.40(9.90–22.67)	11.90 (7.50–17.77)	15.00(10.05–24.25)	5.75(4.60–8.77)	.160	.000	.000
**MO(×10** ^**9**^ **/L)**	0.55(0.45–0.68)	0.61 (0.48–0.79)	0.49(0.32–0.64)	0.32(0.19–0.49)	.054	.000	.517
**MO%**	9.6(7.7–12.1)	10.4 (7.7–12.9)	8.6(5.8–11.5)	4.6(3.7–9.0)	.297	.000	.091
**EO(×10** ^**9**^ **/L)**	0.020(0.000–0.040)	0.015 (0–0.050)	0.000(0.000–0.000)	0.000(0.000–0.0025)	.052	.000	.275
**EO%**	0.3(0.1–0.7)	0.2 (0.1–0.7)	0.1(0.0–0.5)	0.0(0.0–0.1)	.111	.000	.640
**PLT(×10** ^**9**^ **/L)**	194(163–221)	189 (160–225)	161(135–252)	94(76–117)	.993	.000	.001
**CRP (mg/L)**	7.47(3.53–13.92)	7.91 (3.29–14.66)	52.45(34.49–77.21)	69.95(57.57–120.93)	.000	.000	1.0
**NLR**	4.50(2.92–8.13)	6.63 (3.84–10.97)	4.85(2.84–8.05)	15.46(9.27–20.12)	.594	.000	.000
**LCR**	0.125(0.057–0.235)	0.087 (0.051–0.224)	0.016(0.009–0.027)	0.004(0.002–0.006)	.000	.000	.320
**CLR**	7.96(4.24–17.41)	11.49 (4.44–19.58)	59.87(38.26–106.40)	201.76(154.58–355.15)	.000	.000	.320

Note: Data are expressed as medians (P25, P75) or numbers (%).

Abbreviations: IMV, invasive mechanical ventilation; WBC, white blood cell; NE, neutrophil count; NE%, percentage of neutrophils; LY, lymphocyte count; LY%, percentage of lymphocytes; MO, monocyte count; MO%, percentage of monocytes; EO, eosinophil count; EO%, percentage of eosinophils; PLT, platelet count; NLR, neutrophil to lymphocyte ratio; LCR, lymphocyte to CRP ratio; CLR, CRP to lymphocyte ratio.

^1^ Comparison of the total abnormal rate between outpatient and inpatient without IMV COVID-19 groups.

^2^ Comparison of the total abnormal rate between outpatient and inpatient with IMV COVID-19 groups.

^3^ Comparison of the total abnormal rate between inpatient without IMV and inpatient with IMV COVID-19 groups.

### Analysis of routine blood parameters from outpatient COVID-19 and outpatient influenza A groups

Table 1 lists the parameters measured in the routine blood of the outpatient COVID‐19 and outpatient influenza A patients. Analysis showed that the white blood cell count (WBC), neutrophil count (NE), percentage of neutrophils (NE%), monocyte count (MO), percentage of monocytes (MO%), neutrophil to lymphocyte ratio (NLR), and CRP to lymphocyte ratio (CLR) during the early stage of outpatient COVID‐19 were significantly higher than those in the outpatient influenza A group (p<0.05). The lymphocyte count (LY), percentage of lymphocytes (LY%), and lymphocyte to CRP ratio (LCR) in the outpatient COVID‐19 group were significantly lower than those in the outpatient influenza A group (p<0.05).

### Diagnostic efficacy of routine blood parameters between COVID-19 and influenza A

**Fig 1:**
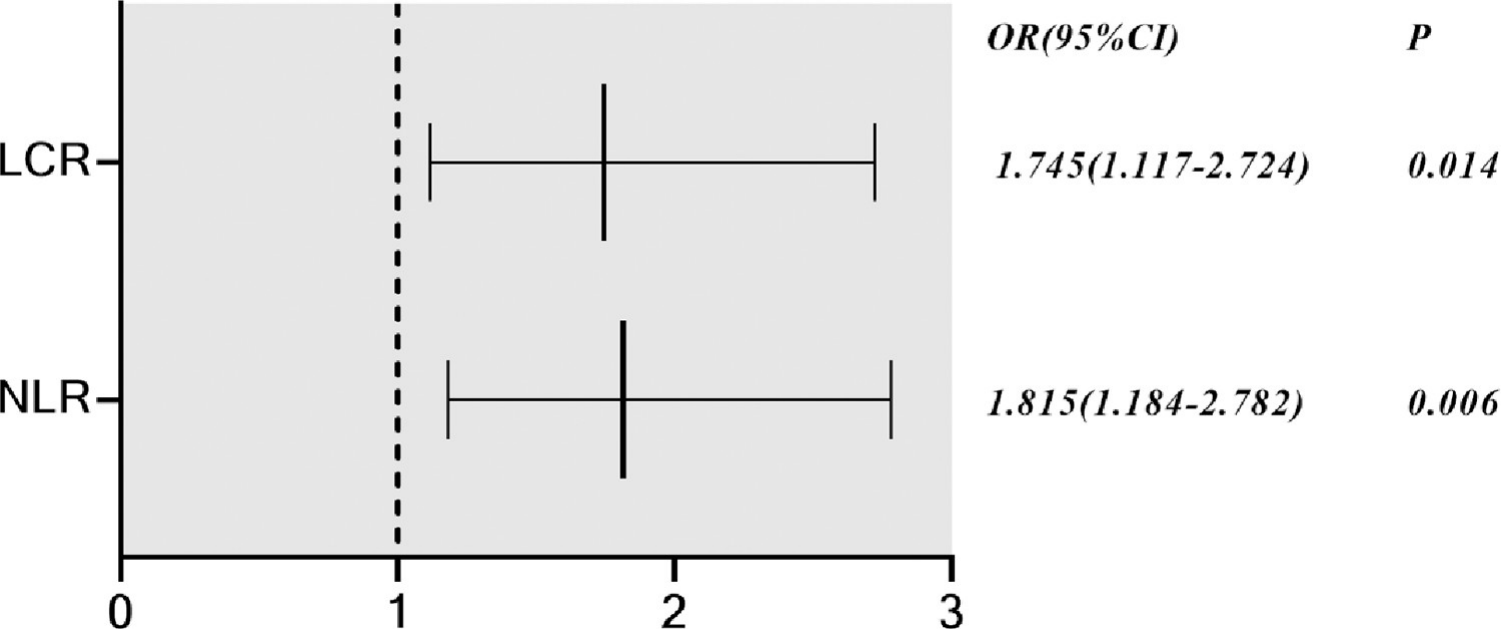
Binary logistic regression analysis of outpatient COVID-19 and influenza A at the first admission of routine blood parameters. NLR, neutrophil to lymphocyte ratio; LCR, lymphocyte to CRP ratio.

**Fig 2:**
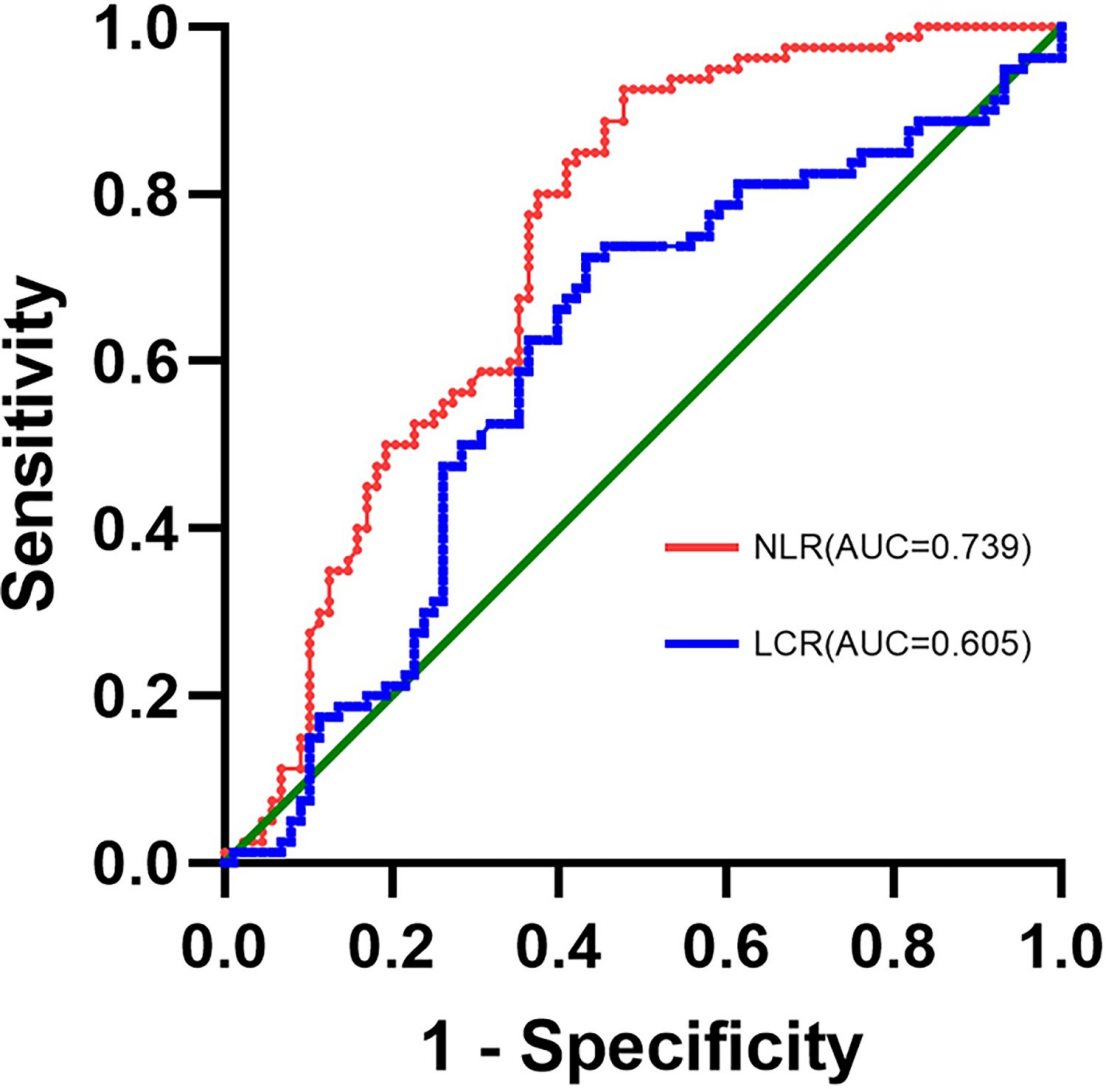
Receiver operating characteristic curve of routine blood parameters in the diagnosis of COVID-19. NLR, neutrophil to lymphocyte ratio; LCR, lymphocyte to CRP ratio. AUC, area under the curve.

Binary logistic regression analysis of statistically significant parameters of routine blood (WBC, NE, NE%, LY, LY%, MO, MO%, NLR, LCR, and CLR), revealed that two variables (NLR and LCR) were independently related to COVID‐19 (Fig 1). The outpatient COVID‐19 group was set as the positive group, and the outpatient influenza A group was set as the negative group. The area under the curve (AUC) of the NLR was 0.739 (95% confidence interval, CI [0.663–0.814], p<0.001), and the AUC of the LCR was 0.605 (95% CI [0.518–0.692], p = 0.019). Based on the ROC curve (Fig 2), the best cut-off point of NLR was found to be 6.48, the diagnostic sensitivity was 52.3%, and the specificity was 92.5%. Therefore, COVID‐19 should be considered when the NLR is greater than 6.48, while influenza A should be considered when the NLR is less than 6.48.

### Analysis of the first routine blood parameters from different COVID-19 groups

Compared with the outpatient COVID‐19 and inpatient without IMV COVID‐19 groups, the inpatient with IMV COVID‐19 group had higher NE% and NLR, and lower LY, LY%, and platelets (PLT) counts (p<0.001). CRP and CLR (CRP to lymphocyte ratio) among the outpatient, inpatient without IMV, and inpatient with IMV COVID-19 groups were (7.91 vs 52.45 vs 69.95) and (11.49 vs 59.87 vs 201.76), respectively. Interestingly, there were no significant differences between the inpatient with IMV and inpatient without IMV COVID-19 groups (p>0.05) (Table 2).

### Establishment of the risk factors for poor COVID-19 prognosis by multiple logistic regression analysis

Multiple logistic regression analysis of statistically significant routine blood parameters (NE%, LY, LY%, PLT, and NLR). According to the comparisons between the outpatient and inpatient with IMV COVID-19 groups, the increase in PLT was found to be associated with an increase in the probability of outpatient COVID-19 (odds ratio, OR, 1.048; 95% CI,1.021–1.076) (p = 0.01). The other parameters (NE%, LY, LY%, and NLR) were not risk factors in the outpatient and inpatient with IMV COVID-19 groups (p>0.05) (Table 3).

**Table 3:** Association between severity and potential risk factors among 123 COVID-19 patients in multiple multinomial logistic regression analysis.

Parameters	Comparisons between outpatient-inpatient with IMV COVID-19 groups			Comparisons between inpatient without IMV-inpatient with IMV COVID-19 groups		
	OR	95%CI	*P*	OR	95%CI	*P*
**NE%**	0.827	0.625–1.094	.657	1.088	0.793–1.493	.600
**LY%**	1.043	0.625–1.742	.871	1.374	0.8–2.361	.250
**LY**	47.083	0.072–30852.011	.244	3.877	0.004–3480.983	.696
**PLT**	1.048	1.021–1.076	.001	1.049	1.021–1.078	.001
**NLR**	1.168	0.934–1.46	.174	0.823	0.549–1.234	.347

Abbreviations: IMV, invasive mechanical ventilation; NE%, percentage of neutrophils; LY, lymphocyte count; LY%, percentage of lymphocytes; PLT, platelet count; NLR, neutrophil to lymphocyte ratio.

According to the comparisons between the inpatient without IMV and inpatient with IMV COVID-19 groups, the increase in PLT was found to be associated with an increase in the probability of inpatient without IMV COVID-19 group (OR, 1.049; 95% CI,1.021–1.078) (p = 0.01). The other parameters (NE%, LY, LY%, and NLR) were not risk factors in the inpatient without IMV and inpatient with IMV COVID-19 groups (p>0.05) (Table 3).

## Discussion

COVID-19 and influenza A can be transmitted through the respiratory system, and ARDS can occur in severe cases. Common clinical manifestations of COVID-19 and influenza A are fever, cough, headache, and other symptoms of upper respiratory tract infection, and both can show decreased WBC, lymphocyte, and eosinophil counts in laboratory tests. However, the two diseases are treated differently, and COVID-19 infected individuals are more likely to develop interstitial lung lesions [5, 6]. Therefore, early and rapid identification of both diseases and their severity is necessary to rationalize limited medical resources (including ICU, invasive mechanical ventilation, etc.), during the COVID-19 and influenza A epidemics.

Our study showed that lymphocytes were lower and the NLR was higher in COVID-19 patients than in patients with influenza A. In particular, the NLR was significantly elevated in inpatients with IMV COVID-19.

Elevated NLR is most often seen in advanced cancer [7], autoimmune diseases [8], and infections [9]. The NLR is a biomarker that combines two aspects of the immune system: the innate immune response, mainly due to neutrophils [10], and adaptive immunity, by lymphocytes [11]. Studies have shown that lymphocyte count decreases after COVID-19 infection, and COVID-19 is more likely to cause lymphopenia than other respiratory viruses [12–14]. Pontelli et al. showed that COVID-19 effectively infects PBMCs and high levels of active caspase 3/7 (a marker of apoptosis) were found in lymphocytes [15]. A recent meta-analysis has shown that the NLR is associated with COVID-19 disease progression and mortality [16]. In our study, the NLR differed between COVID-19 and influenza A, which was useful in differentiating between the two diseases.

As a part of the immune system, platelets work with innate immune cells (such as neutrophils and macrophages) to capture and kill pathogens. Platelets contain a variety of immune receptors that allow them to act as sentinels to recognize intravascular pathogens [17]. Thrombocytopenia is another feature of COVID-19. In a retrospective study of 1476 hospitalized COVID-19 patients, 20.7% were found to have thrombocytopenia and thrombocytopenia was associated with mortality [18]. Bhanu Kanth Manne et al. found that mRNA from the SARS-CoV-2 N1 gene was detected in platelets from COVID-19 patients [19]. In our study, we found a significant reduction in PLT in the first blood test in inpatients with IMV COVID-19, and PLT can be used as one of the indicators for stratified treatment of COVID-19.

This study also has some limitations. First, this was a retrospective study with a small sample size, and subsequent expansion of the sample size is needed to obtain appropriate NLR cut-off values to better differentiate COVID-19 from influenza A. Second, the patients with COVID-19 in this study were not tested for viral strains, and different strains may have different clinical manifestations and laboratory test characteristics.

## Conclusions

NLR and PLT are routine and inexpensive biomarkers. Our study found that the NLR can differentiate between COVID-19 and influenza A during COVID-19 and influenza A epidemics, which is higher in COVID-19 with IMV. The decrease in PLT is a danger signal of poor prognosis of COVID-19.

## Supporting information

S1 Checklist STROBE statement—checklist of items that should be included in reports of observational studies. (DOCX)

## References

[1] Guan WJ, Ni ZY, Hu Y, Liang WH, Ou CQ, He JX, et al. Clinical Characteristics of Coronavirus Disease 2019 in China. N Engl J Med. 2020;382(18):1708–20. Epub 20200228. doi: 10.1056/NEJMoa2002032 . 32109013 PMC7092819

[2] Bautista Edgar, Chotpitayasunondh Tawee, Gao Zhancheng, Scott A Harper Michael Shaw, Timothy M Uyeki, et al. Clinical aspects of pandemic 2009 influenza A (H1N1) virus infection. N Engl J Med. 2010;362(18):1708–19. doi: 10.1056/NEJMra1000449 . 20445182

[3] Abbasi J. Combining Rapid PCR and Antibody Tests Improved COVID-19 Diagnosis. JAMA. 2020;324:1386. doi: 10.1001/jama.2020.19129 . 33048137

[4] Mathur R, Rentsch CT, Morton CE, Hulme WJ, Schultze A, MacKenna B, et al. Ethnic differences in SARS-CoV-2 infection and COVID-19-related hospitalisation, intensive care unit admission, and death in 17 million adults in England: an observational cohort study using the OpenSAFELY platform. Lancet. 2021;397(10286):1711–24. Epub 20210430. doi: 10.1016/S0140-6736(21)00634-6 . 33939953 PMC8087292

[5] Li Y, Wang H, Wang F, Du H, Liu X, Chen P, et al. Comparison of hospitalized patients with pneumonia caused by COVID-19 and influenza A in children under 5 years. Int J Infect Dis. 2020;98:80–3. Epub 20200612. doi: 10.1016/j.ijid.2020.06.026 . 32535301 PMC7289729

[6] Zhang JJ, Dong X, Cao YY, Yuan YD, Yang YB, Yan YQ, et al. Clinical characteristics of 140 patients infected with SARS-CoV-2 in Wuhan, China. Allergy. 2020;75(7):1730–41. Epub 20200227. doi: 10.1111/all.14238 . 32077115

[7] Grenader T, Waddell T, Peckitt C, Oates J, Starling N, Cunningham D, et al. Prognostic value of neutrophil-to-lymphocyte ratio in advanced oesophago-gastric cancer: exploratory analysis of the REAL-2 trial. Ann Oncol. 2016;27(4):687–92. Epub 20160119. doi: 10.1093/annonc/mdw012 . 26787231

[8] Ma L, Zeng A, Chen B, Chen Y, Zhou R. Neutrophil to lymphocyte ratio and platelet to lymphocyte ratio in patients with systemic lupus erythematosus and their correlation with activity: A meta-analysis. International Immunopharmacology. 2019;76. doi: 10.1016/j.intimp.2019.105949 . 31634817

[9] Cataudella E, Giraffa CM, Di Marca S, Pulvirenti A, Alaimo S, Pisano M, et al. Neutrophil-To-Lymphocyte Ratio: An Emerging Marker Predicting Prognosis in Elderly Adults with Community-Acquired Pneumonia. J Am Geriatr Soc. 2017;65(8):1796–801. Epub 20170413. doi: 10.1111/jgs.14894 . 28407209

[10] Bardoel BW, Kenny EF, Sollberger G, Zychlinsky A. The balancing act of neutrophils. Cell Host Microbe. 2014;15(5):526–36. doi: 10.1016/j.chom.2014.04.011 . 24832448

[11] Cooper MD, Alder MN. The evolution of adaptive immune systems. Cell. 2006;124(4):815–22. doi: 10.1016/j.cell.2006.02.001 . 16497590

[12] Jia Z, Yan X, Gao L, Ding S, Bai Y, Zheng Y, et al. Comparison of Clinical Characteristics Among COVID-19 and Non-COVID-19 Pediatric Pneumonias: A Multicenter Cross-Sectional Study. Front Cell Infect Microbiol. 2021;11:663884. Epub 20210701. doi: 10.3389/fcimb.2021.663884 . 34277466 PMC8281119

[13] Lippi G, Plebani M. Laboratory abnormalities in patients with COVID-2019 infection. Clin Chem Lab Med. 2020;58(7):1131–4. doi: 10.1515/cclm-2020-0198 . 32119647

[14] Huang C, Wang Y, Li X, Ren L, Zhao J, Hu Y, et al. Clinical features of patients infected with 2019 novel coronavirus in Wuhan, China. Lancet. 2020;395(10223):497–506. Epub 20200124. doi: 10.1016/S0140-6736(20)30183-5 . 31986264 PMC7159299

[15] Pontelli MC, Castro IA, Martins RB, La Serra L, Veras FP, Nascimento DC, et al. SARS-CoV-2 productively infects primary human immune system cells in vitro and in COVID-19 patients. J Mol Cell Biol. 2022;14(4). doi: 10.1093/jmcb/mjac021 . 35451490 PMC9384834

[16] Zinellu A, Mangoni AA. A systematic review and meta-analysis of the association between the neutrophil, lymphocyte, and platelet count, neutrophil-to-lymphocyte ratio, and platelet-to-lymphocyte ratio and COVID-19 progression and mortality. Expert Rev Clin Immunol. 2022;18(11):1187–202. Epub 20220905. doi: 10.1080/1744666X.2022.2120472 . 36047369

[17] Portier I, Campbell RA. Role of Platelets in Detection and Regulation of Infection. Arterioscler Thromb Vasc Biol. 2021;41(1):70–8. Epub 20201029. doi: 10.1161/ATVBAHA.120.314645 . 33115274 PMC7770024

[18] Yang X, Yang Q, Wang Y, Wu Y, Xu J, Yu Y, et al. Thrombocytopenia and its association with mortality in patients with COVID-19. J Thromb Haemost. 2020;13:161. doi: 10.1111/jth.14848 . 32302435 PMC9906135

[19] Manne BK, Denorme F, Middleton EA, Portier I, Rowley JW, Stubben C, et al. Platelet gene expression and function in patients with COVID-19. Blood. 2020;136(11):1317–29. doi: 10.1182/blood.2020007214 . 32573711 PMC7483430

